# Supplementary material for: HNF4A defines tissue-specific circadian rhythms by beaconing BMAL1::CLOCK chromatin binding and shaping the rhythmic chromatin landscape
Source: Nat Commun. 2021 Nov 3;12:6350. doi: 10.1038/s41467-021-26567-3 (PMC8566521; doi:10.1038/s41467-021-26567-3)
Supplement: Supplementary file 1 — Supplementary Information [file 41467_2021_26567_MOESM1_ESM.pdf]

**Supplementary Information**

# HNF4A defines tissue-specific circadian rhythms by beaconing BMAL1::CLOCK chromatin binding and shaping the rhythmic chromatin landscape

Meng Qu[1,4,5], Han Qu[2,4], Zhenyu Jia[2,3], Steve A. Kay[1,5]

[1] Department of Neurology, Keck School of Medicine, University of Southern California, Los Angeles, CA, 90089, United States

[2] Department of Botany and Plant Sciences, University of California, Riverside, CA, 92521, United States

[3] Graduate Program in Genetics, Genomics, and Bioinformatics, University of California, Riverside, CA, 92521, United States

[4] These authors contributed equally: Meng Qu, Han Qu.

[5] Corresponding Authors: Meng Qu, Ph.D. (mengqu@usc.edu); Steve A. Kay, Ph.D. (stevekay@usc.edu).

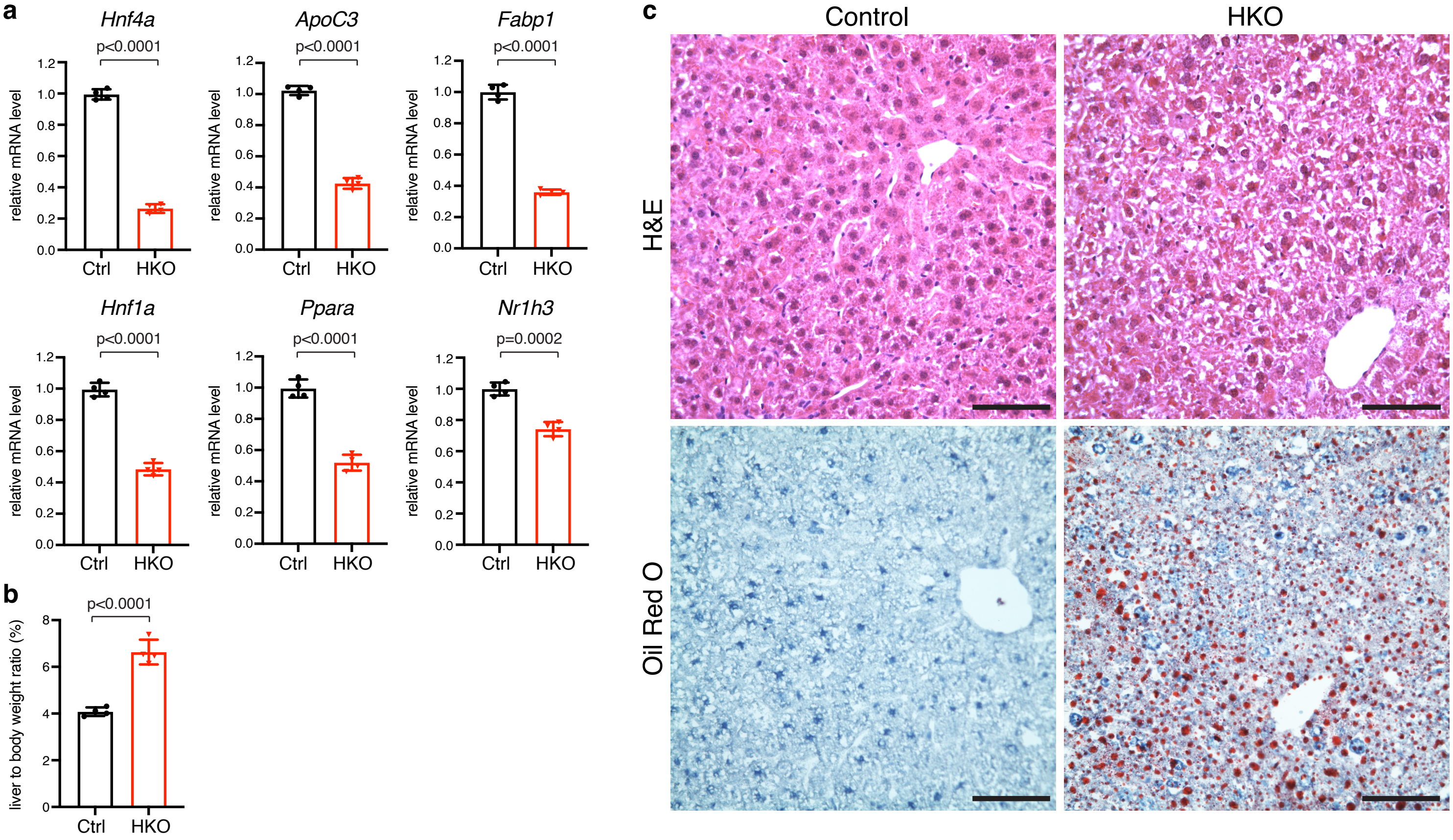


**Supplementary Fig. 1. Characterization of liver-specific *Hnf4a* knockout mice (*Hnf4a*$^{fl/fl}$ *Alb-Cre*$^{+/-}$ *Per2-luc*$^{+/+}$) at 10-11 weeks of age.**
**(a)** Transcript level of genes was determined by RT-qPCR using liver samples isolated from control or HKO mice at ZT18. Displayed are the means ± SD (n = 4) normalized to *Rplp0* expression levels. Statistical significance was determined by two-tailed Student's t-test. **(b)** Liver to body weight ratio of control or HKO liver (means ± SD, n = 4). Statistical significance was determined by two-tailed Student's t-test. **(c)** Histopathological analysis of the *Hnf4a* knockout livers. Representative images of H&E staining (upper panel) and Oil Red O staining (lower panel). Paraffin-embedded liver sections were used for H&E staining and frozen sections from the same samples were used for Oil Red O staining. All images are 400X. Bar, 100 μm.

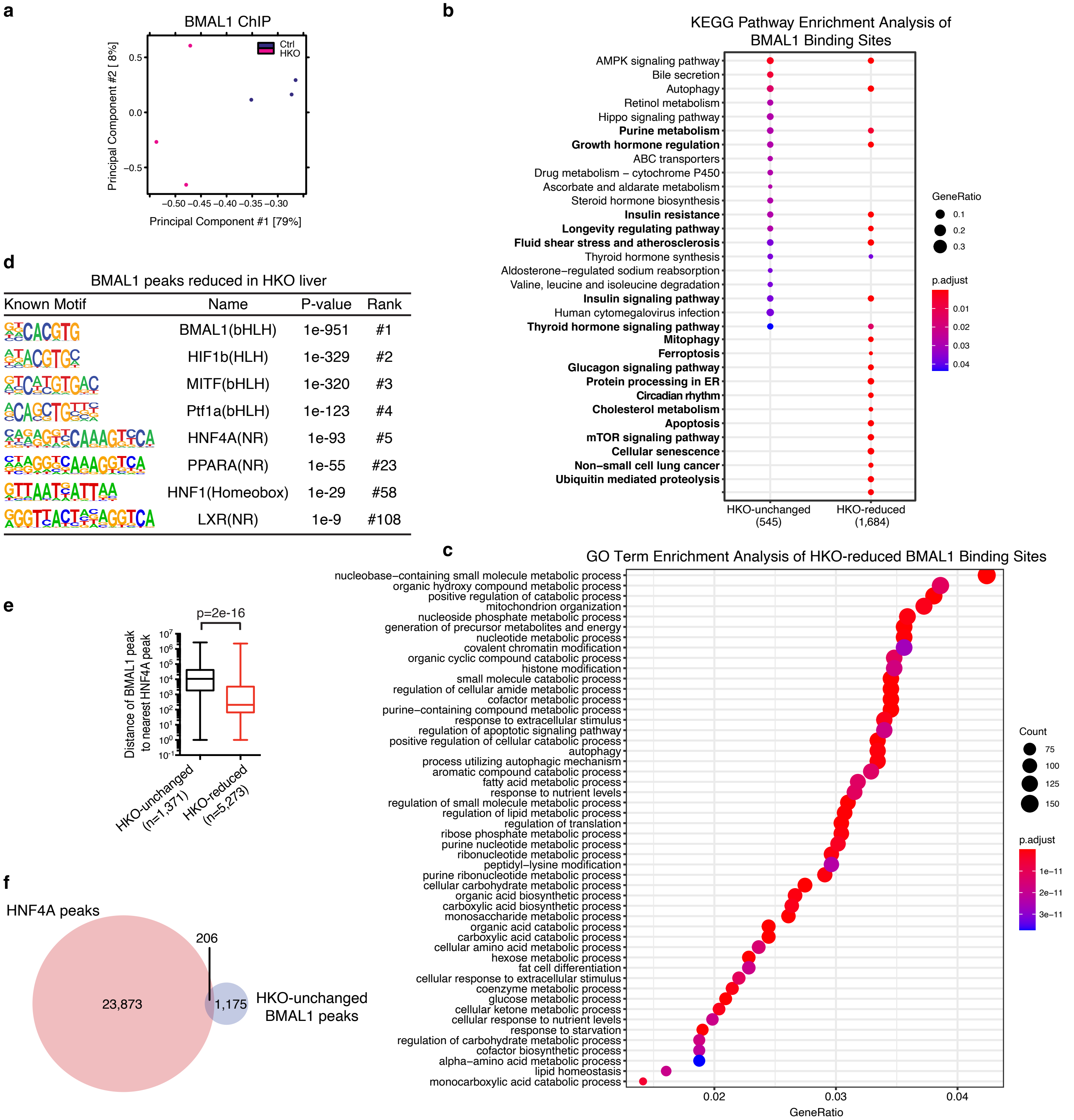


**Supplementary Fig. 2. BMAL1 chromatin binding is attenuated in the *Hnf4a* knockout liver.**
**(a)** PCA plot of BMAL1 ChIP-seq counts at ZT6 across consensus BMAL1 peaks in control and HKO liver. **(b)** KEGG pathway enrichment analyses of HKO-unchanged or reduced BMAL1 binding genes. **(c)** Gene ontology (GO) (“biological process” sub-ontology) terms associated with HKO-reduced BMAL1 binding sites. **(d)** Motif analysis of HKO-deprived BMAL1 binding sites defined in Fig. 1b. Known consensus motifs are shown with corresponding enrichment significance values. **(e)** Base pair unit distance from each BMAL1 peak to the closest HNF4A peak was calculated and box-plotted. Center line, median; box limits, 25th and 75th percentile; whiskers, 1.5x interquartile range. Statistical significance was determined by two-tailed Student’s t-test. **(f)** Venn diagram showing overlap between BMAL1-binding sites that were not significantly changed in HKO (at ZT6) and all HNF4A binding sites (at ZT16).

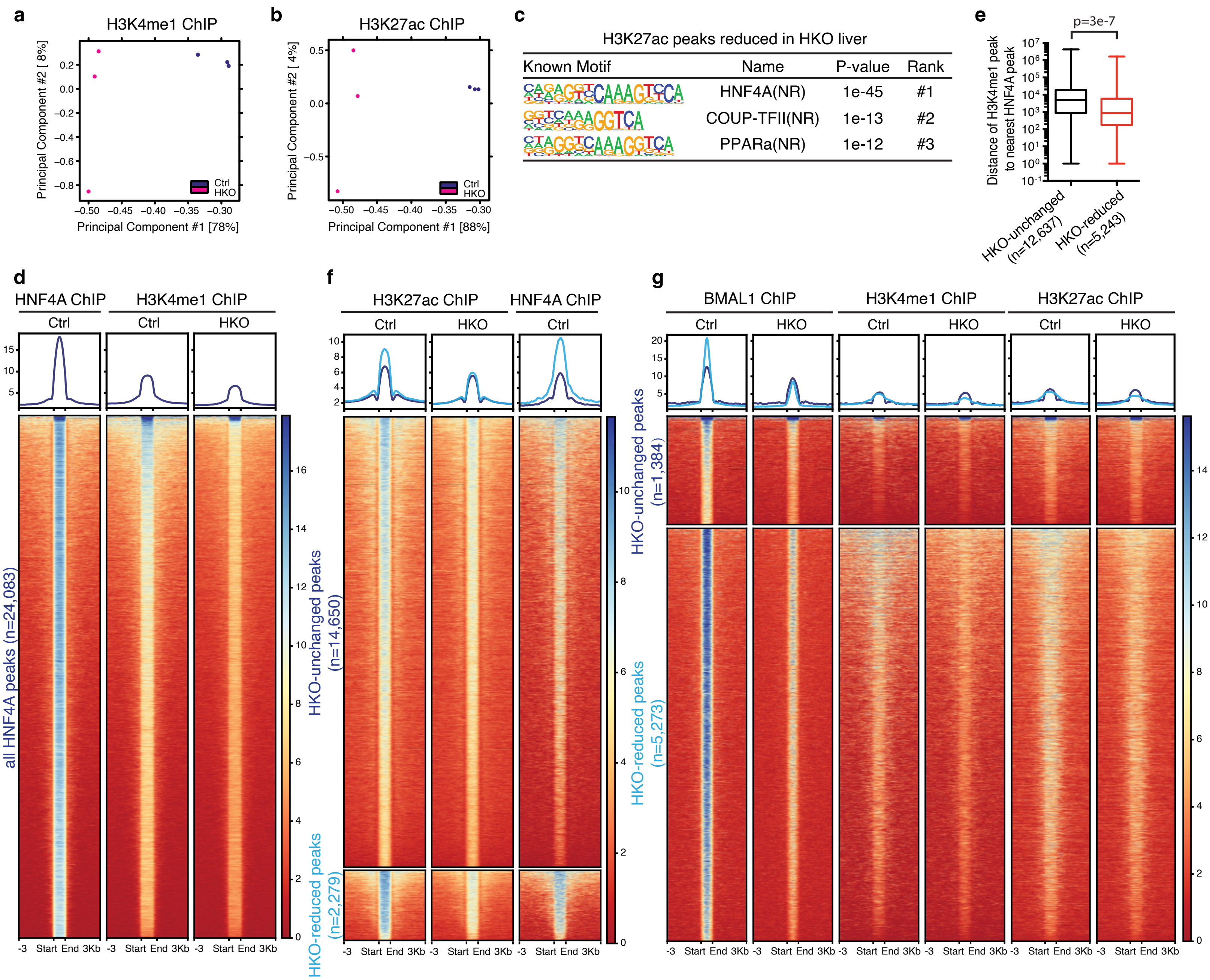


**Supplementary Fig. 3. *Hnf4a* knockout alters the genome-wide epigenetic landscape.**
**(a-b)** PCA plot of H3K4me1 **(a)** or H3K27ac **(b)** ChIP-seq counts at ZT6 across consensus peaks in control and HKO liver. **(c)** Motif analysis of HKO-deprived H3K27ac sites. Known consensus motifs are shown with corresponding enrichment significance values. **(d)** H3K4me1 occupancy in control or HKO liver was plotted at each HNF4A binding site (at ZT16). Each horizontal line represents a single HNF4A binding site. **(e)** Base pair unit distance from each H3K4me1 peak to the closest HNF4A peak was calculated and box-plotted. Center line, median; box limits, 25th and 75th percentile; whiskers, 1.5x interquartile range. Statistical significance was determined by two-tailed Student's t-test. **(f)** H3K27ac peaks in control and HKO livers were partitioned into three categories with DiffBind (the HKO-enriched group has only 3 peaks and couldn't be plotted), and then the corresponding HNF4A occupancy (at ZT16) at each H3K27ac site was plotted. Each horizontal line represents a single H3K27ac site. Peaks were ordered vertically by strength of H3K27ac ChIP signal in control liver. **(g)** BMAL1 peaks in control and HKO livers were partitioned into three categories with DiffBind (the HKO-enriched group has only 3 peaks and couldn't be plotted), and then the corresponding H3K4me1 or H3K27ac occupancy (at ZT6) at each BMAL1 binding site was plotted. Each horizontal line represents a single BMAL1 binding site. Peaks were ordered vertically by strength of BMAL1 ChIP signal in control liver.

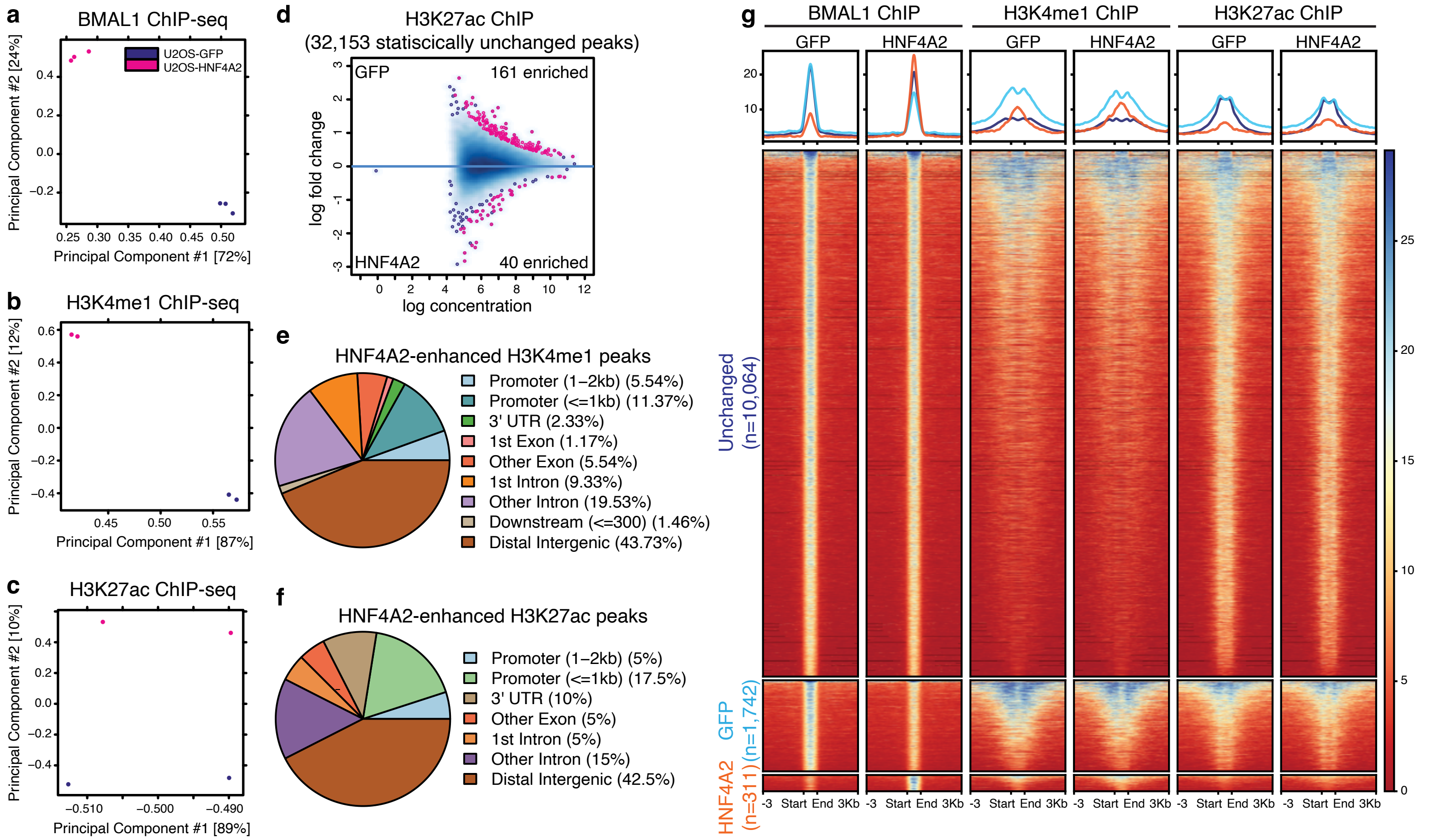


**Supplementary Fig. 4. Ectopic HNF4A2 expression reprograms epigenetic landscape and induces tissue-specific BMAL1 bindings.**
**(a-c)** PCA plot of BMAL1 **(a)**, H3K4me1 **(b)**, or H3K27ac **(c)** ChIP-seq counts across consensus peaks in U2OS-GFP and U2OS-HNF4A2 cells. **(d)** MA plot showing differential H3K27ac occupancy in U2OS-GFP and U2OS-HNF4A2 cells, using threshold of FDR < 0.05. The x-axis represents the mean number of reads (log scaled) within the peaks across all samples. The y-axis represents the log fold change between the two samples. **(e-f)** Distribution of genomic annotations of HNF4A2-induced H3K4me1 **(e)** or H3K27ac **(f)** sites. **(g)** BMAL1 peaks in U2OS-GFP and U2OS-HNF4A2 cells were partitioned into three categories with DiffBind, and then the corresponding H3K4me1 and H3K27ac occupancy at each BMAL1 binding site was plotted. Each horizontal line represents a single BMAL1 binding site. Peaks were ordered vertically by strength of BMAL1 ChIP signal.

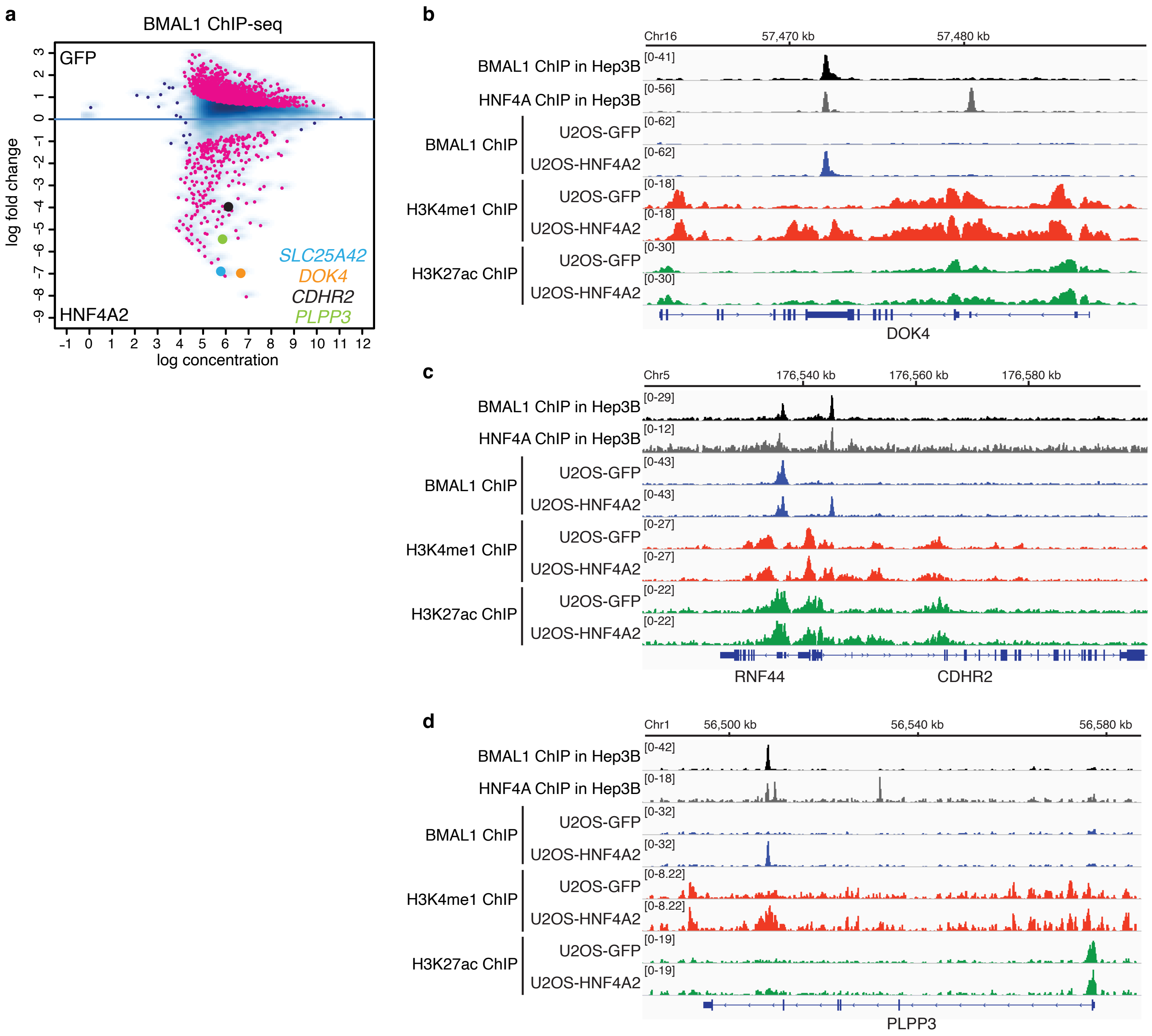


**Supplementary Fig. 5. Representative genome tracks showing HNF4A2-induced BMAL1 peaks and locally enhanced H3K4me1 and H3K27ac signals.**
**(a)** Representative BMAL1 binding events significantly induced by HNF4A2 are highlighted in MA plot. **(b-d)** IGV genome tracks showing BMAL1, HNF4A, H3K4me1, and H3K27ac enrichment at the *DOK4* **(b)**, *CDHR2* **(c)**, and *PLPP3* **(d)** gene loci in the indicated cells, based on normalized ChIP-seq read coverage. Track heights are indicated.

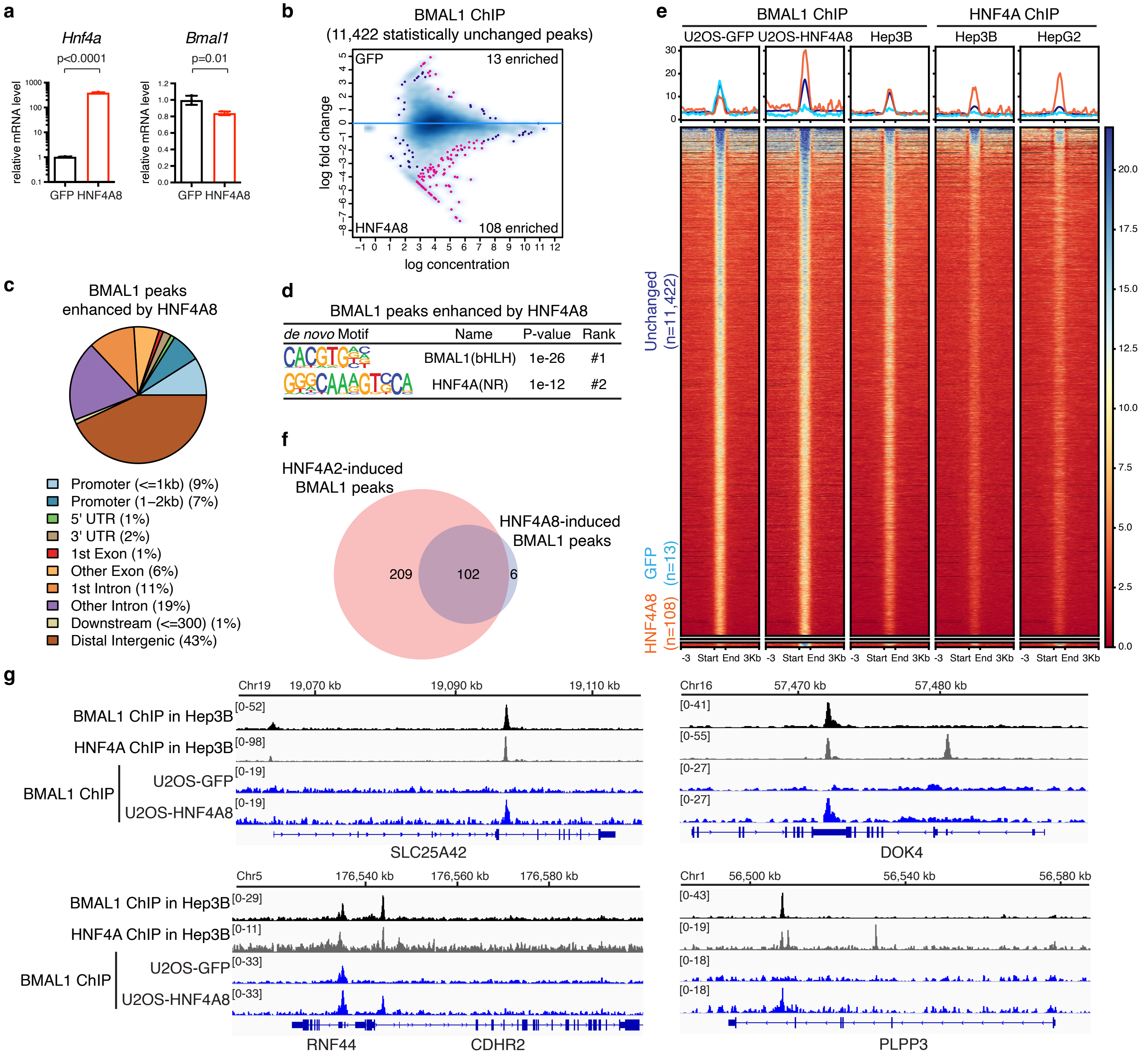


**Supplementary Fig. 6. The fetal isoform HNF4A8 is capable of inducing BMAL1 genome binding.** **(a)** Transcript level of genes was determined by RT-qPCR. Displayed are means ± SD (n = 3 cell culture wells) normalized to *Rplp0* expression levels. Statistical significance was determined by two-tailed Student's t-test. **(b)** MA plot showing differential BMAL1 occupancy, using threshold of FDR < 0.1. The x-axis represents the mean number of reads (log scaled) within the peaks across all samples. The y-axis represents the log fold change between the two samples. **(c)** Distribution of genomic annotations of HNF4A8-enhanced BMAL1 peaks. **(d)** Motif analysis of HNF4A8-enhanced BMAL1 binding sites. *de novo* consensus motifs are shown with corresponding enrichment significance values. **(e)** BMAL1 peaks in U2OS-GFP and U2OS-HNF4A8 cells were partitioned into three categories with DiffBind. Then the corresponding BMAL1 and HNF4A occupancy in Hep3B or HepG2 cells were plotted by centering at each BMAL1 binding site in U2OS cells. Each horizontal line represents a single BMAL1 binding site in U2OS. Peaks were ordered vertically by strength of BMAL1 ChIP signal in U2OS. **(f)** Venn diagram showing overlap between BMAL1-binding sites induced by HNF4A2 or HNF4A8. **(g)** IGV genome tracks showing BMAL1 and HNF4A enrichment at the *SLC25A42*, *DOK4*, *CDHR2*, and *PLPP3* gene loci in the indicated cells, based on normalized ChIP-seq read coverage. Track heights are indicated.

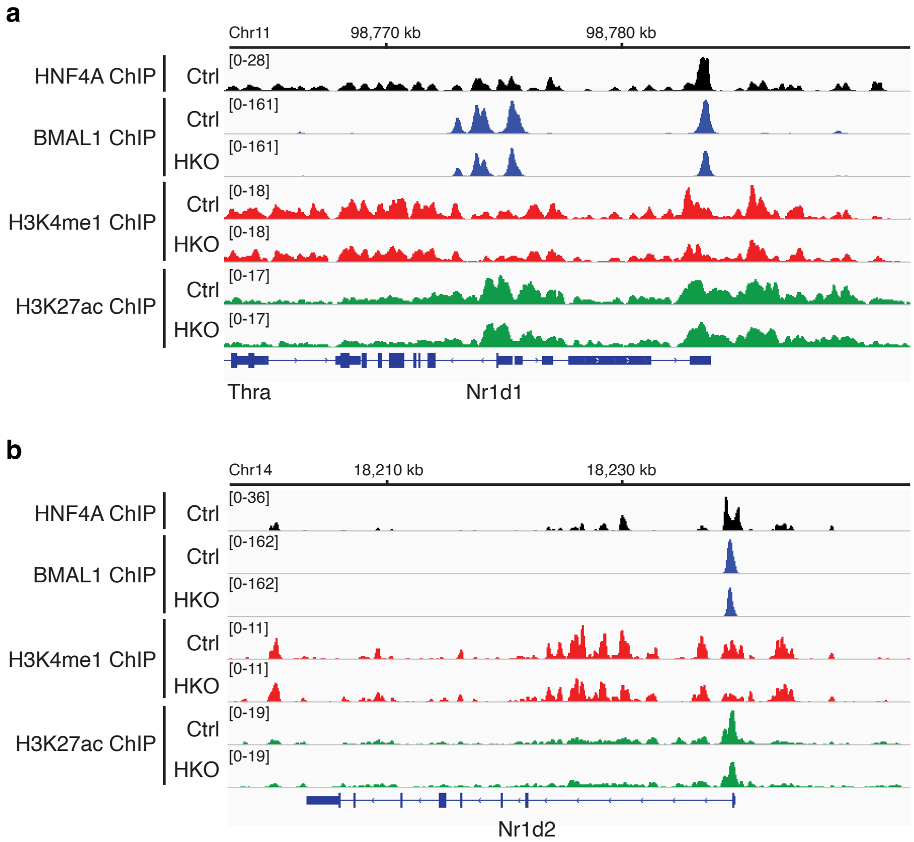


**Supplementary Fig. 7. Genome tracks showing HKO-reduced BMAL1 peaks and locally decreased H3K4me1 and H3K27ac signals at BMAL1::CLOCK-dependent genes.**
IGV genome tracks of HNF4A (at ZT16), BMAL1 (at ZT6), H3K4me1 (at ZT6), and H3K27ac (at ZT6) enrichment at *Nr1d1* **(a)** and *Nr1d2* **(b)** gene loci in the indicated genotypes, based on normalized ChIP-seq read coverage. Track heights are indicated.

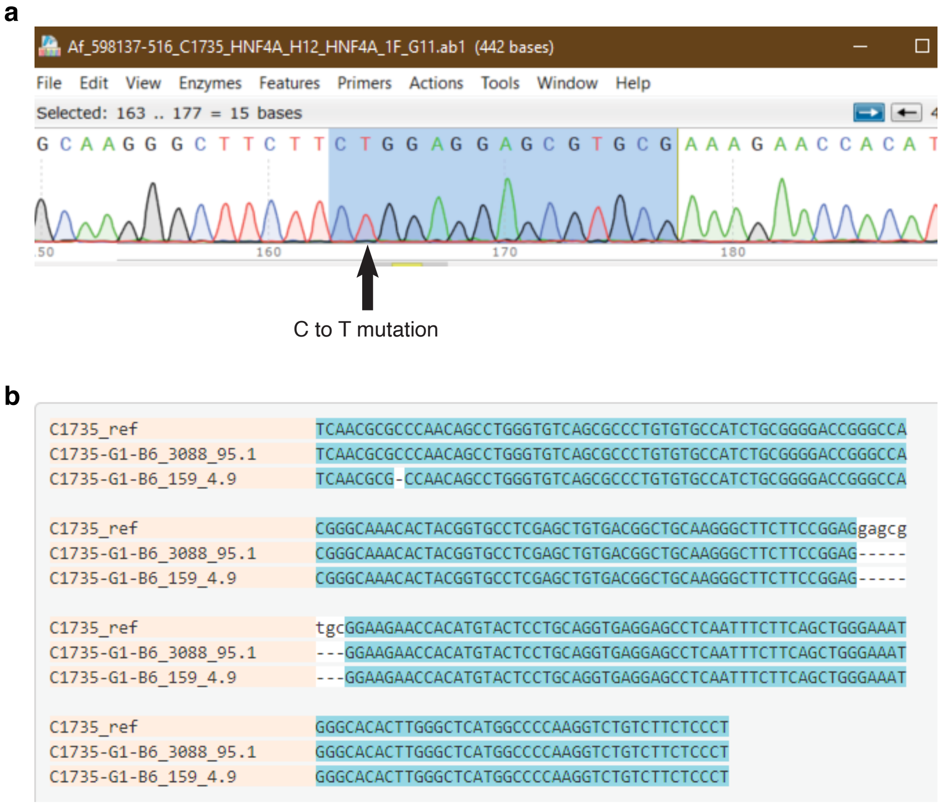


**Supplementary Fig. 8. Homozygous *Hnf4a* knockout and R85W mutation were generated in Hep3B cells using CRISPR-CAS9.**
**(a)** Homozygous *HNF4A-R85W* mutant line was validated by Sanger sequencing. **(b)** Homozygous *Hnf4a* knockout line was validated by NGS.

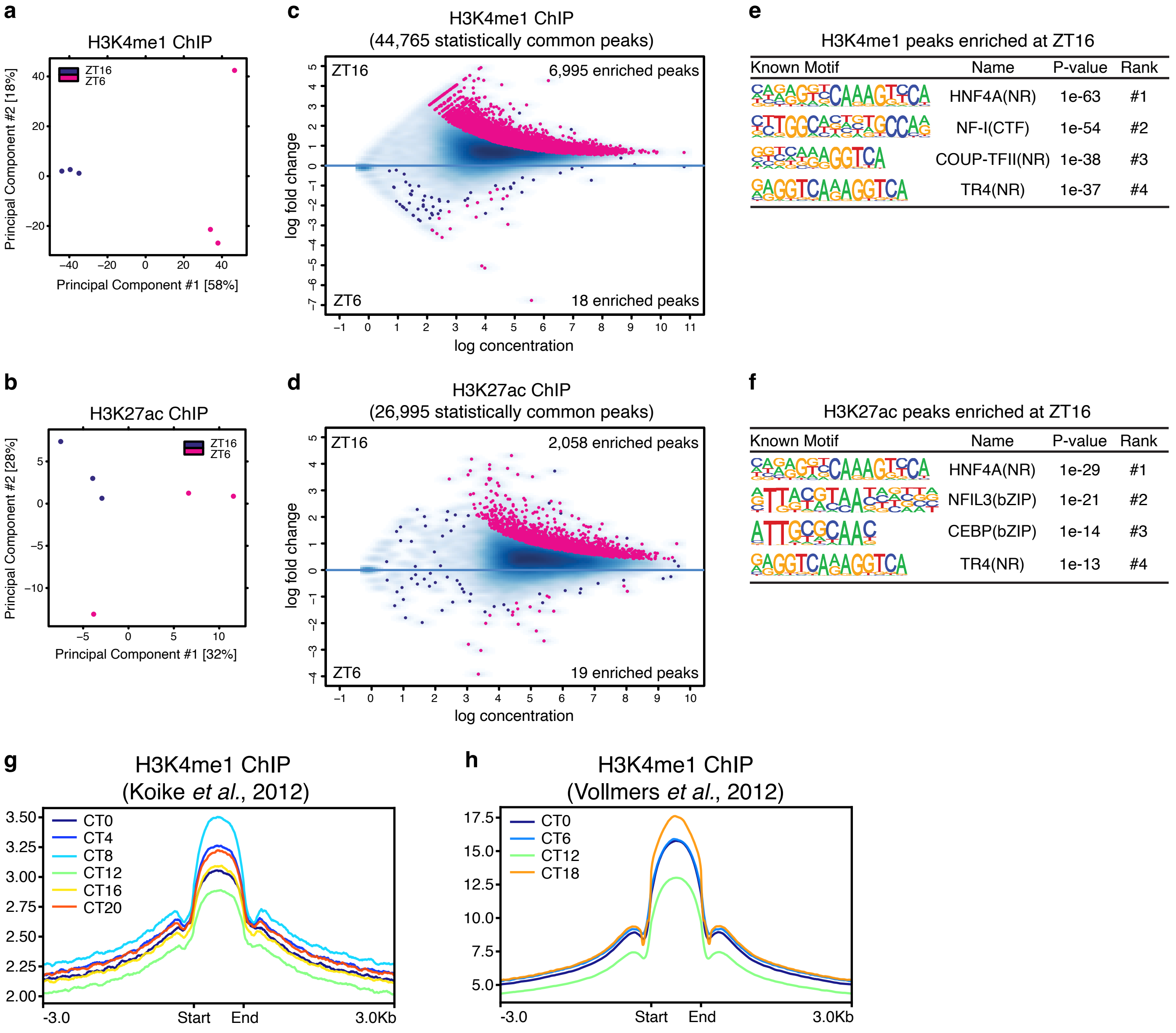


**Supplementary Fig. 9. Mouse liver chromatin is more accessible at night, synchronized with HNF4A recruitment.**

**(a-b)** PCA plot of H3K4me1 **(a)** or H3K27ac **(b)** ChIP-seq counts across consensus peaks at ZT16 and ZT6. **(c-d)** MA plot showing differential H3K4me1 **(c)** or H3K27ac **(d)** signals at ZT16 and ZT6, using threshold of FDR $< 0.05$. The x-axis represents the mean number of reads (log scaled) within the peaks across all samples. The y-axis represents the log fold change between the two samples. **(e-f)** Motif analysis of ZT16-enriched H3K4me1 **(e)** or H3K27ac sites **(f)** that were defined in (c) or (d). Known consensus motifs are shown with corresponding enrichment significance values. **(g-h)** Circadian rhythms of H3K4me1 deposition characterized in literature PMID 22936566 **(g)** and PMID 23217262 **(h)**. Profiles of genome-wide H3K4me1 distribution throughout the day were plotted using DeepTools (v3.3.0).

**a**

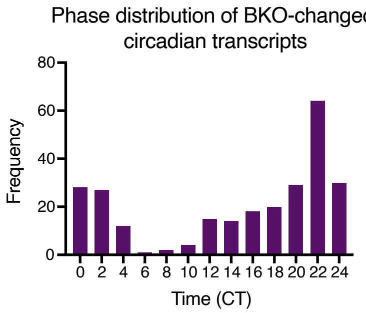


**b**

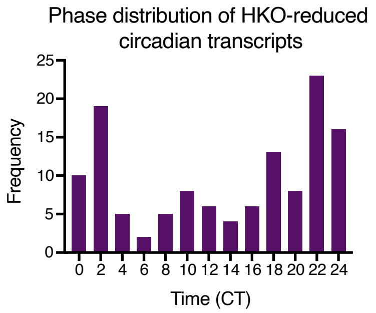


**c**

| Circadian genes differentially expressed in *Bmal1* knockout liver | | |
|---|---|---|
| **GO term** | **# Genes** | **FDR** |
| lipid metabolic process | 15 | 4.10E-02 |
| triglyceride metabolic process | 5 | 4.10E-02 |
| cholesterol metabolic process | 7 | 4.10E-02 |
| fatty acid metabolic process | 8 | 9.00E-02 |
| ADP biosynthetic process | 3 | 9.00E-02 |
| oxidation-reduction process | 16 | 9.60E-02 |
| glutathione metabolic process | 5 | 9.60E-02 |
| liver development | 6 | 1.30E-01 |
| nucleobase-containing compound metabolic process | 4 | 2.10E-01 |
| cellular response to lithium ion | 3 | 5.30E-01 |
| negative regulation of cell-cell adhesion | 3 | 5.60E-01 |
| L-cysteine catabolic process | 2 | 8.40E-01 |
| response to insulin | 4 | 9.90E-01 |
| circadian sleep/wake cycle | 2 | 9.90E-01 |
| response to cadmium ion | 3 | 9.90E-01 |

**d**

| Circadian genes downregulated in *Hnf4a* knockout liver | | |
|---|---|---|
| **GO term** | **# Genes** | **FDR** |
| lipid metabolic process | 14 | 2.40E-03 |
| steroid metabolic process | 7 | 3.20E-03 |
| cholesterol metabolic process | 7 | 3.20E-03 |
| oxidation-reduction process | 14 | 2.60E-02 |
| circadian rhythm | 5 | 3.00E-01 |
| fatty acid metabolic process | 5 | 8.00E-01 |
| response to interleukin-18 | 2 | 8.70E-01 |
| acute-phase response | 3 | 8.70E-01 |
| liver development | 4 | 8.70E-01 |
| triglyceride metabolic process | 3 | 9.00E-01 |
| kynurenine metabolic process | 2 | 9.00E-01 |
| negative regulation of peptidase activity | 4 | 9.20E-01 |
| amyloid precursor protein catabolic process | 2 | 9.20E-01 |
| L-serine metabolic process | 2 | 9.20E-01 |
| tryptophan catabolic process to kynurenine | 2 | 9.60E-01 |

**e**

| Circadian genes differentially expressed in *Bmal1* knockout liver | | |
|---|---|---|
| **KEGG pathway** | **# Genes** | **FDR** |
| Glyoxylate and dicarboxylate metabolism | 6 | 1.50E-03 |
| Metabolic pathways | 31 | 4.10E-03 |
| Glycerolipid metabolism | 5 | 1.80E-01 |
| Carbon metabolism | 6 | 2.30E-01 |
| Biosynthesis of amino acids | 5 | 2.30E-01 |
| PPAR signaling pathway | 5 | 2.40E-01 |
| Peroxisome | 5 | 2.40E-01 |
| Tryptophan metabolism | 4 | 2.40E-01 |
| alpha-Linolenic acid metabolism | 3 | 4.40E-01 |
| Drug metabolism - cytochrome P450 | 4 | 4.60E-01 |
| Fat digestion and absorption | 3 | 7.40E-01 |

**f**

| Circadian genes downregulated in *Hnf4a* knockout liver | | |
|---|---|---|
| **KEGG pathway** | **# Genes** | **FDR** |
| Metabolic pathways | 27 | 1.60E-04 |
| Steroid biosynthesis | 4 | 2.30E-02 |
| Tryptophan metabolism | 5 | 2.30E-02 |
| PPAR signaling pathway | 4 | 8.70E-01 |
| Fat digestion and absorption | 3 | 1.00E+00 |
| Glycerolipid metabolism | 3 | 1.00E+00 |
| Synthesis and degradation of ketone bodies | 2 | 1.00E+00 |
| Hepatitis C | 4 | 1.00E+00 |

**Supplementary Fig. 10. Transcripts most altered in *Hnf4a* or *Bmal1* knockout liver tend to be rhythmically expressed.**
**(a-b)** Phase distribution of circadian transcripts most altered in *Bmal1* **(a)** or *Hnf4a* **(b)** knockout liver. **(c-d)** GO terms ("biological process" sub-ontology) associated with circadian transcripts most altered in *Bmal1* **(c)** or *Hnf4a* **(d)** knockout liver were determined by DAVID (https://david.ncifcrf.gov/home.jsp). **(e-f)** KEGG functional pathways associated with circadian transcripts most altered in *Bmal1* **(e)** or *Hnf4a* **(f)** knockout liver were determined by DAVID.

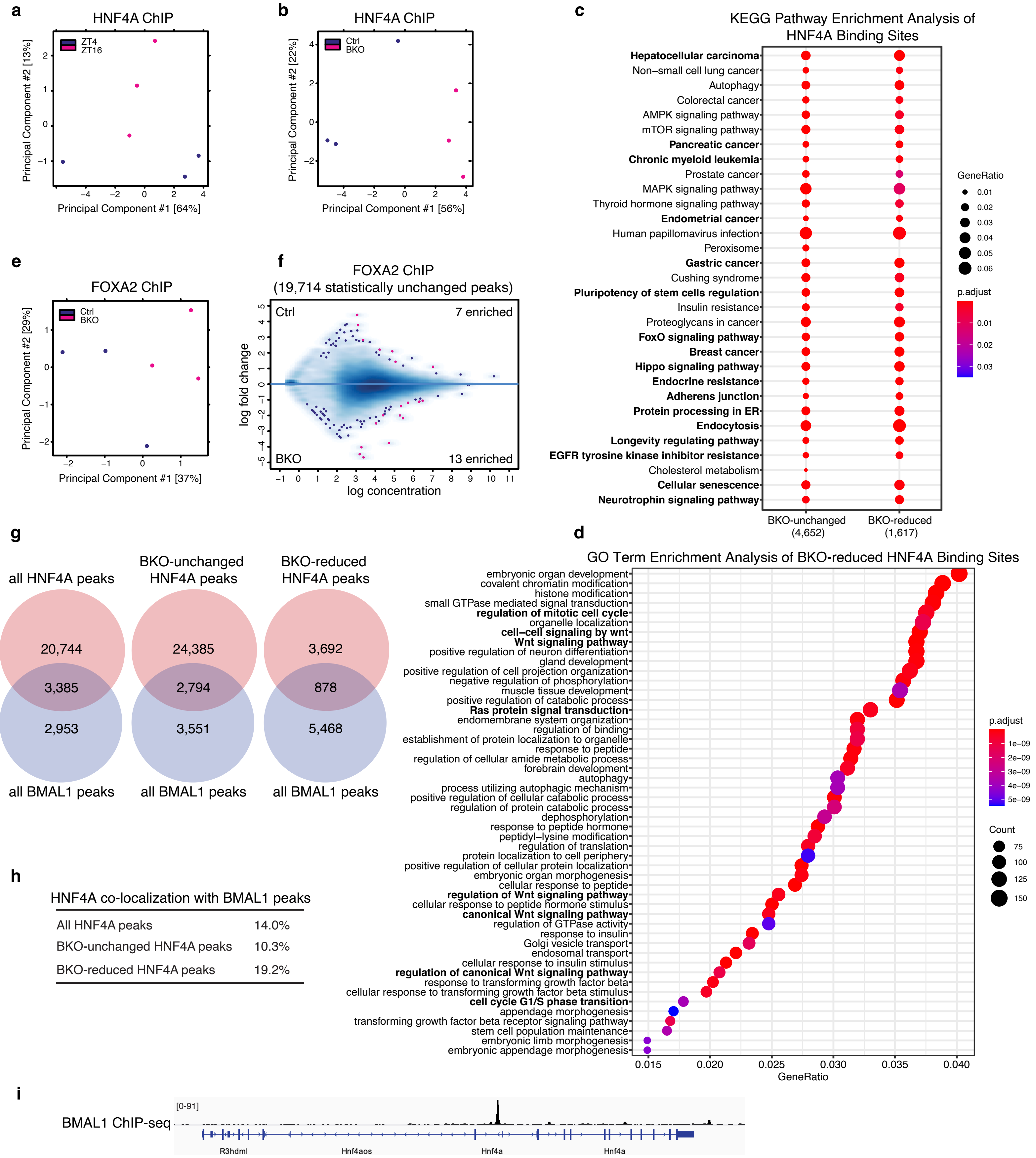
a
HNF4A ChIP
ZT4
ZT16
Principal Component #1 [64%]
Principal Component #2 [13%]
b
HNF4A ChIP
Ctrl
BKO
Principal Component #1 [56%]
Principal Component #2 [22%]
c
KEGG Pathway Enrichment Analysis of HNF4A Binding Sites
Hepatocellular carcinoma
Non−small cell lung cancer
Autophagy
Colorectal cancer
AMPK signaling pathway
mTOR signaling pathway
Pancreatic cancer
Chronic myeloid leukemia
Prostate cancer
MAPK signaling pathway
Thyroid hormone signaling pathway
Endometrial cancer
Human papillomavirus infection
Peroxisome
Gastric cancer
Cushing syndrome
Pluripotency of stem cells regulation
Insulin resistance
Proteoglycans in cancer
FoxO signaling pathway
Breast cancer
Hippo signaling pathway
Endocrine resistance
Adherens junction
Protein processing in ER
Endocytosis
Longevity regulating pathway
EGFR tyrosine kinase inhibitor resistance
Cholesterol metabolism
Cellular senescence
Neurotrophin signaling pathway
BKO-unchanged (4,652)
BKO-reduced (1,617)
GeneRatio
0.01
0.02
0.03
0.04
0.05
0.06
p.adjust
0.01
0.02
0.03
e
FOXA2 ChIP
Ctrl
BKO
Principal Component #1 [37%]
Principal Component #2 [29%]
f
FOXA2 ChIP
(19,714 statistically unchanged peaks)
Ctrl
7 enriched
BKO
13 enriched
log fold change
log concentration
g
all HNF4A peaks
BKO-unchanged HNF4A peaks
BKO-reduced HNF4A peaks
20,744
3,385
2,953
24,385
2,794
3,551
3,692
878
5,468
all BMAL1 peaks
all BMAL1 peaks
all BMAL1 peaks
h
HNF4A co-localization with BMAL1 peaks
All HNF4A peaks 14.0%
BKO-unchanged HNF4A peaks 10.3%
BKO-reduced HNF4A peaks 19.2%
d
GO Term Enrichment Analysis of BKO-reduced HNF4A Binding Sites
embryonic organ development
covalent chromatin modification
histone modification
small GTPase mediated signal transduction
regulation of mitotic cell cycle
organelle localization
cell−cell signaling by wnt
Wnt signaling pathway
positive regulation of neuron differentiation
gland development
positive regulation of cell projection organization
negative regulation of phosphorylation
muscle tissue development
positive regulation of catabolic process
Ras protein signal transduction
endomembrane system organization
regulation of binding
establishment of protein localization to organelle
response to peptide
regulation of cellular amide metabolic process
forebrain development
autophagy
process utilizing autophagic mechanism
positive regulation of cellular catabolic process
regulation of protein catabolic process
dephosphorylation
response to peptide hormone
peptidyl−lysine modification
regulation of translation
protein localization to cell periphery
positive regulation of cellular protein localization
embryonic organ morphogenesis
cellular response to peptide
regulation of Wnt signaling pathway
cellular response to peptide hormone stimulus
canonical Wnt signaling pathway
regulation of GTPase activity
response to insulin
Golgi vesicle transport
endosomal transport
cellular response to insulin stimulus
regulation of canonical Wnt signaling pathway
response to transforming growth factor beta
cellular response to transforming growth factor beta stimulus
cell cycle G1/S phase transition
appendage morphogenesis
transforming growth factor beta receptor signaling pathway
stem cell population maintenance
embryonic limb morphogenesis
embryonic appendage morphogenesis
GeneRatio
p.adjust
1e−09
2e−09
3e−09
4e−09
5e−09
Count
75
100
125
150
i
BMAL1 ChIP-seq
[0-91]
R3hdml
Hnf4aos
Hnf4a
Hnf4a

**Supplementary Fig. 11. The circadian clock modulates genome-wide DNA binding of HNF4A.**
**(a)** PCA plot of HNF4A ChIP-seq counts across consensus HNF4A peaks at ZT4 and ZT16 after chronic jet lag. **(b)** PCA plot of HNF4A ChIP-seq counts at ZT16 across consensus peaks in control and BKO liver. **(c)** KEGG pathway enrichment analyses of BKO-unchanged or reduced HNF4A binding genes. **(d)** Gene ontology (GO) ("biological process" sub-ontology) terms associated with BKO-reduced HNF4A binding sites. **(e)** PCA plot of FOXA2 ChIP-seq counts at ZT16 across consensus peaks in control and BKO liver. **(f)** MA plot showing differential FOXA2 peaks in control and BKO livers, using threshold of FDR $< 0.05$. The x-axis represents the mean number of reads (log scaled) within the peaks across all samples. The y-axis represents the log fold change between the two samples. **(g-h)** Venn diagram **(g)** and table **(h)** showing overlap between BMAL1 (at ZT6) and HNF4A (at ZT16) peaks. **(i)** IGV genome track showing BMAL1 enrichment at the *Hnf4a* gene at ZT6 in the control mouse liver.

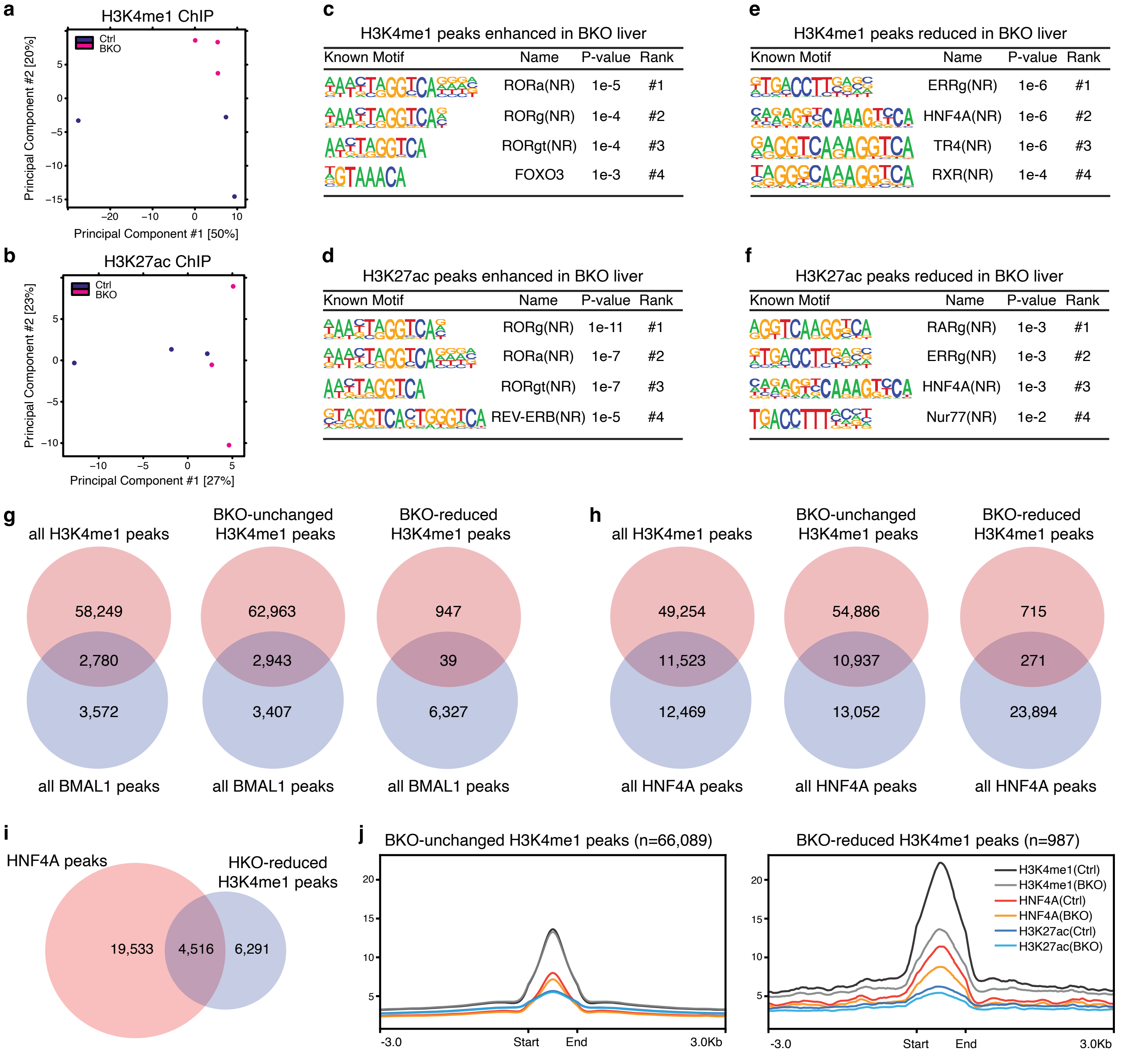


**Supplementary Fig. 12. *Bmal1* knockout alters epigenetic landscape in the liver, seemingly due to attenuated HNF4A activity.**

**(a-b)** PCA plot of H3K4me1 **(a)** or H3K27ac **(b)** ChIP-seq counts at ZT16 across consensus peaks in control and BKO liver. **(c-f)** Motif analysis of BKO-reduced/BKO-enhanced H3K4me1 or H3K27ac sites defined in Fig. 7c-d. Known consensus motifs are shown with corresponding enrichment significance values. **(g)** Venn diagram showing overlap between H3K4me1 (at ZT16) and BMAL1 peaks (at ZT6). **(h)** Venn diagram showing overlap between H3K4me1 and HNF4A peaks (both at ZT16). **(i)** Venn diagram showing overlap between H3K4me1 sites significantly reduced in HKO (at ZT6) and all HNF4A binding sites (at ZT16).

**(j)** Metaplot showing average intensity of H3K4me1, HNF4A, and H3K27ac ChIP-seq signals (all at ZT16) in control or BKO livers surrounding BKO-unchanged (left panel) or BKO-reduced (right panel) H3K4me1 peak centers.
