# Supplementary material for: HNF4A defines tissue-specific circadian rhythms by beaconing BMAL1::CLOCK chromatin binding and shaping the rhythmic chromatin landscape
Source: Nat Commun. 2021 Nov 3;12:6350. doi: 10.1038/s41467-021-26567-3 (PMC8566521; doi:10.1038/s41467-021-26567-3)
Supplement: Supplementary file 3 — Description of Additional Supplementary Files [file 41467_2021_26567_MOESM3_ESM.pdf]

<u>Descriptions of Additional Supplementary Files</u>

File Name: Supplementary Data 1
Description: List of HKOreduced BMAL1 binding sites

File Name: Supplementary Data 2
Description: List of HKOreduced or BKO-changed transcripts showing circadian expression in CircaDB

File Name: Supplementary Data 3
Description: List of BKOreduced HNF4A binding sites
