# Supplementary material for: HNF4A defines tissue-specific circadian rhythms by beaconing BMAL1::CLOCK chromatin binding and shaping the rhythmic chromatin landscape
Source: Nat Commun. 2021 Nov 3;12:6350. doi: 10.1038/s41467-021-26567-3 (PMC8566521; doi:10.1038/s41467-021-26567-3)
Supplement: Supplementary file 7 — Reporting Summary [file 41467_2021_26567_MOESM7_ESM.pdf]

nature research

Corresponding author(s): Meng Qu, Steve A. Kay

Last updated by author(s): Sep 24, 2021

Reporting Summary

Nature Research wishes to improve the reproducibility of the work that we publish. This form provides structure for consistency and transparency in reporting. For further information on Nature Research policies, see our Editorial Policies and the Editorial Policy Checklist.

Statistics

For all statistical analyses, confirm that the following items are present in the figure legend, table legend, main text, or Methods section.

n/a	Confirmed	
☐	☒	The exact sample size (*n*) for each experimental group/condition, given as a discrete number and unit of measurement
☐	☒	A statement on whether measurements were taken from distinct samples or whether the same sample was measured repeatedly
☐	☒	The statistical test(s) used AND whether they are one- or two-sided<br>*Only common tests should be described solely by name; describe more complex techniques in the Methods section.*
☐	☒	A description of all covariates tested
☒	☐	A description of any assumptions or corrections, such as tests of normality and adjustment for multiple comparisons
☐	☒	A full description of the statistical parameters including central tendency (e.g. means) or other basic estimates (e.g. regression coefficient) AND variation (e.g. standard deviation) or associated estimates of uncertainty (e.g. confidence intervals)
☐	☒	For null hypothesis testing, the test statistic (e.g. *F*, *t*, *r*) with confidence intervals, effect sizes, degrees of freedom and *P* value noted<br>*Give P values as exact values whenever suitable.*
☒	☐	For Bayesian analysis, information on the choice of priors and Markov chain Monte Carlo settings
☒	☐	For hierarchical and complex designs, identification of the appropriate level for tests and full reporting of outcomes
☒	☐	Estimates of effect sizes (e.g. Cohen's *d*, Pearson's *r*), indicating how they were calculated

Our web collection on statistics for biologists contains articles on many of the points above.

Software and code

Policy information about availability of computer code

Data collection	The circadian period was calculated using the LumiCycle software (Actimetrics, version 3.002).
Data analysis	Trimmomatic (v0.36) was used to trim NGS reads.<br>Bowtie2 (v2.3.4.1) was used to align reads to genome.<br>SAMtools (v1.10) was used for BAM file processing and merging of replicate BAM files.<br>PicardTools (v2.18.3) was used for PCR duplicates removal.<br>MACS2 (v2.1.2) was used for peak calling.<br>DeepTools (v3.3.0) was used to generate bigWig track coverage files, heatmaps, and metaplots.<br>DiffBind (v3.2.7) was used to make PCA plots and call differential peaks.<br>HOMER (v4.11.1) was used to identify overlapping binding loci of two transcription factors, enriched motifs, and annotation of genomic features.<br>PrimerQuest (https://www.idtdna.com/PrimerQuest) was used to design RT-qPCR primers.

For manuscripts utilizing custom algorithms or software that are central to the research but not yet described in published literature, software must be made available to editors and reviewers. We strongly encourage code deposition in a community repository (e.g. GitHub). See the Nature Research guidelines for submitting code & software for further information.

Data

Policy information about availability of data

All manuscripts must include a data availability statement. This statement should provide the following information, where applicable:

- Accession codes, unique identifiers, or web links for publicly available datasets
- A list of figures that have associated raw data
- A description of any restrictions on data availability

Raw data (fastq files) and final processed data (bigWig and peak files) for NGS experiments are available on GEO through accession number GSE157452 [https://www.ncbi.nlm.nih.gov/geo/query/acc.cgi?acc=GSE157452]. Figures associated with raw data are Fig. 1-7. GSE35262 [https://www.ncbi.nlm.nih.gov/geo/query/acc.cgi?acc=GSE35262] and E-MTAB-941 [https://www.ebi.ac.uk/arrayexpress/experiments/E-MTAB-941/] were used to analyze PPARA, HNF1A, and LXR deposition at BMAL1 binding sites. GSE39860 [https://www.ncbi.nlm.nih.gov/geo/query/acc.cgi?acc=GSE39860] and SRA025656 [https://www.ncbi.nlm.nih.gov/sra/?term=SRA025656] were used for reanalysis of H3K4me1 circadian rhythms. CircaDB [http://circadb.hogeneschlab.org/] was used for identification of circadian transcripts. Source data are provided with this paper.

Field-specific reporting

Please select the one below that is the best fit for your research. If you are not sure, read the appropriate sections before making your selection.

☒ Life sciences ☐ Behavioural & social sciences ☐ Ecological, evolutionary & environmental sciences

For a reference copy of the document with all sections, see nature.com/documents/nr-reporting-summary-flat.pdf

Life sciences study design

All studies must disclose on these points even when the disclosure is negative.

Sample size	By convention and in line with our other studies (PMID: 30530698), we used 2 or 3 biological replicates for NGS experiments, 3-4 biological replicates for RT-qPCR assays, and 4-5 biological replicates for circadian period calculations.
Data exclusions	No data were excluded from the analyses.
Replication	All experiments used replicates which substantially agreed with each other.
Randomization	Control or knockout mice were randomly allocated into the experiments after their genotypes were determined. In the liver histology experiments, for each tissue sample we examined 3 or 4 randomly selected imaging areas.
Blinding	The investigators were not blinded during data collection (wet lab work) because blinding in not a common practice in the area of molecular biology. However, authors in charge of data analysis were blinded to the identity of experimental samples.

Reporting for specific materials, systems and methods

We require information from authors about some types of materials, experimental systems and methods used in many studies. Here, indicate whether each material, system or method listed is relevant to your study. If you are not sure if a list item applies to your research, read the appropriate section before selecting a response.

Materials & experimental systems

n/a	Involved in the study	
☐	☒	Antibodies
☐	☒	Eukaryotic cell lines
☒	☐	Palaeontology and archaeology
☐	☒	Animals and other organisms
☒	☐	Human research participants
☒	☐	Clinical data
☒	☐	Dual use research of concern

Methods

n/a	Involved in the study	
☐	☒	ChIP-seq
☒	☐	Flow cytometry
☒	☐	MRI-based neuroimaging

Antibodies

Antibodies used	Antibodies used in the western blots are anti-BMAL1 (Cell signaling, #14020, Clone number D2L7G, 1:1000), anti-HNF4A (Abcam, ab181604, Clone number EPR16885, 1:1000), and anti-TUBULIN (Sigma-Aldrich, T0198, Clone number D66, 1:1000). Antibodies used in the ChIP-seq experiments are anti-HNF4A (Abcam, ab41898, Clone number K9218), anti-BMAL1 (Cell Signaling, #14020, Clone number D2L7G), anti-H3K4me1 (Abcam, ab8895), anti-H3K27ac (Abcam, ab4729), and anti-FOXA2 (Abcam, ab256493, Clone number EPR22919-71).

Validation	All antibodies used in the western blots have been validated by the manufacturers, stating that the antibodies recognize endogenous levels of the target proteins in mouse tissues.<br>For all antibodies used in the ChIP-seq experiments, the manufacturers state that they recognize endogenous target proteins in both mouse tissues and human cell cultures and are suitable for ChIP-seq assays. Besides, our results demonstrated their strong activities.

Eukaryotic cell lines

Policy information about cell lines

Cell line source(s)	The Hep3B cell line was a gift from Dr. Michael Karin at UCSD, originally purchased from ATCC. HEK 293T, U2OS, and HepG2 cell lines were directly purchased from ATCC.
Authentication	All experiments in this study used low-passage cell cultures. The morphology of each cell line was consistent with images and descriptions on the ATCC website. RT-qPCR of liver cancer cell lines (Hep3B and HepG2) indicated high expression level of liver-specific factors.
Mycoplasma contamination	All cell lines were periodically confirmed to be mycoplasma-free by using the PlasmoTest mycoplasma detection kit (InvivoGen #rep-pt1).
Commonly misidentified lines<br>(See ICLAC register)	No commonly misidentified cell lines were used in the study.

Animals and other organisms

Policy information about studies involving animals; ARRIVE guidelines recommended for reporting animal research

Laboratory animals	All animal care and experiments were performed under the institutional protocols approved by the Institutional Animal Care and Use Committee (IACUC, #20826) at the University of Southern California. Hnf4a floxed mice (The Jackson Library #004665) were crossed with Albumin-Cre mice (The Jackson Library #003574) and Per2-luciferase reporter mice (The Jackson Library #006852) to obtain Hnf4afl/+;Alb-Cre+/-;Per2-luc+/+ and Hnf4afl/fl;Alb-Cre-/-;Per2-luc+/+ mice, which were then mated to obtain Hnf4afl/fl;Alb-Cre +/-;Per2-luc+/+ (HKO) and Hnf4afl/fl;Alb-Cre-/-;Per2-luc+/+ (Control) littermates. Bmal1 floxed mice (The Jackson Library #007668) were crossed with mice expressing Albumin-Cre (The Jackson Library #003574) to obtain Arntlfl/+;Alb-Cre+/- and Arntlfl/fl;Alb-Cre-/- mice which were then mated to obtain Arntlfl/fl;Alb-Cre+/- (BKO) and Arntlfl/fl;Alb-Cre-/- (Control) littermates. In all experiments, male mice between 10 and 12 wk of age were used. In all experiments except the jet lag treatment, mice were housed in a room with controlled temperature of 21–23°C and humidity of 35–40% under a 12-h light/12-h dark (LD) cycle with free access to food and water. The chronic jet lag treatment was performed by housing experimental mice in the light-tight circadian cabinet and switching lighting conditions between two light onset schedules which are apart by 8 hours every three days from 7 to 11 wk of age.
Wild animals	The study did not involve wild animals.
Field-collected samples	The study did not involve samples collected from the field.
Ethics oversight	All animal care and experiments were performed under the institutional protocols approved by the Institutional Animal Care and Use Committee (IACUC) at the University of Southern California.

Note that full information on the approval of the study protocol must also be provided in the manuscript.

ChIP-seq

Data deposition

- [x] Confirm that both raw and final processed data have been deposited in a public database such as GEO.
- [x] Confirm that you have deposited or provided access to graph files (e.g. BED files) for the called peaks.

Data access links<br>*May remain private before publication.*	https://www.ncbi.nlm.nih.gov/geo/query/acc.cgi?acc=GSE157452<br>Reviewer token: sjoncewstvgzhgf
Files in database submission	The database includes raw data (fastq files) and processed data (bigWig and BED files) for all NGS studies.
Genome browser session<br>(e.g. UCSC)	No longer applicable

Methodology

Replicates	All ChIP-seq experiments used two or thee biological replicates. For ChIP-seq with mouse tissues, replicates were liver samples isolated from different mice. For ChIP-seq with cell lines, replicates were cells from different cell culture dishes. The replicates were agreeable with one another.
Sequencing depth	For each experiment, the total reads number was >25 million. >90% of the reads were uniquely mapped to the mouse or human genome. The reads were 150 bp long, single-end.
Antibodies	Antibodies used in the ChIP-seq experiments are anti-HNF4A (Abcam, ab41898), anti-BMAL1 (Cell Signaling, #14020), anti-H3K4me1

	(Abcam, ab8895), anti-H3K27ac (Abcam, ab4729), and anti-FOXA2 (Abcam, ab256493).
Peak calling parameters	Single-end ChIP-seq reads were trimmed using Trimmomatic (v0.36) and then aligned to hg38 or mm10 genome with Bowtie2 (v2.3.4.1). BAM files were processed using SAMtools (v1.10) and PCR duplicates were removed with PicardTools (v2.18.3). Peaks were called in MACS2 (v2.1.2) using default settings and IgG mock ChIP-seq files for normalization. To call peaks, HNF4A ChIP-seq used q-value of 10e-8, and the other ChIP-seqs used q-value of 0.05.
Data quality	Quality of raw data was evaluated by FastQC. Peaks were called in MACS2 (v2.1.2) using q-value (FDR) of 10e-8 for HNF4A ChIP-seq and 0.05 for the other ChIP-seqs. The peak numbers (that have been specified in the figures) were in line with results from prior studies. The peaks called were further validated by visualizing the peak postion files (e.g. BED and narrowPeak) and bigWig files for all samples including the mock IP control in a genome browser. Also, legacy data was downloaded and analyzed as important controls for validation of peak positions.
Software	Trimmomatic (v0.36) was used to trim ChIP-seq reads.<br>Bowtie2 (v2.3.4.1) was used to align reads to genome.<br>SAMtools (v1.10) was used for BAM file processing and merging of replicate BAM files.<br>PicardTools (v2.18.3) was used for PCR duplicates removal.<br>MACS2 (v2.1.2) was used for peak calling.<br>DeepTools (v3.3.0) was used to generate bigWig track coverage files, heatmaps, and metaplots.<br>DiffBind was used to make PCA plots and call differential peaks.<br>HOMER (v4.11.1) was used to identify overlapping binding loci of two transcription factors, enriched motifs, and annotation of genomic features.
