# Supplementary figures and images for: HNF4A defines tissue-specific circadian rhythms by beaconing BMAL1::CLOCK chromatin binding and shaping the rhythmic chromatin landscape
Source: Nat Commun. 2021 Nov 3;12:6350. doi: 10.1038/s41467-021-26567-3 (PMC8566521; doi:10.1038/s41467-021-26567-3)

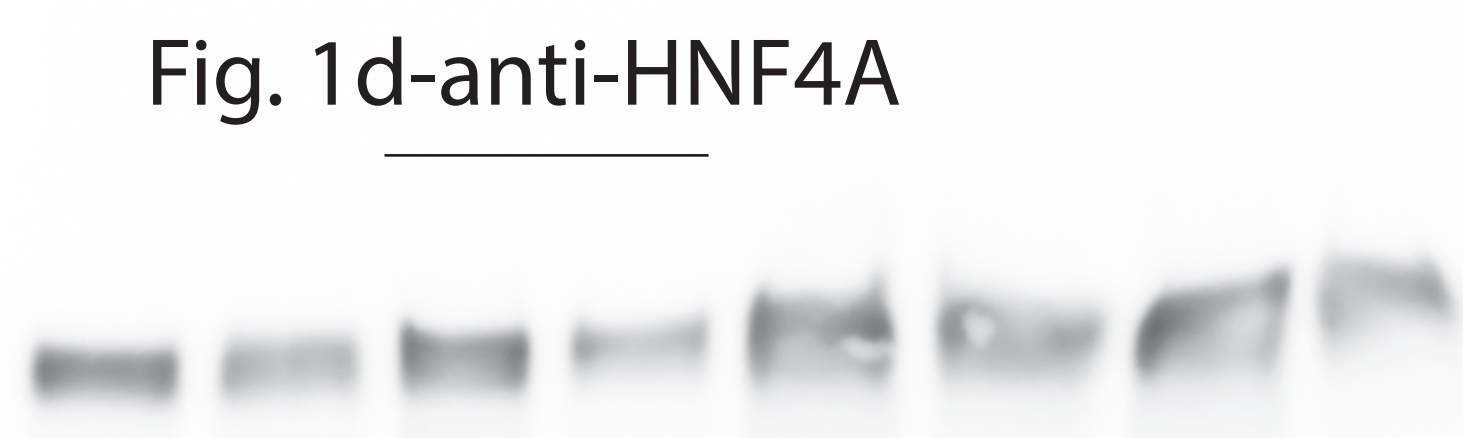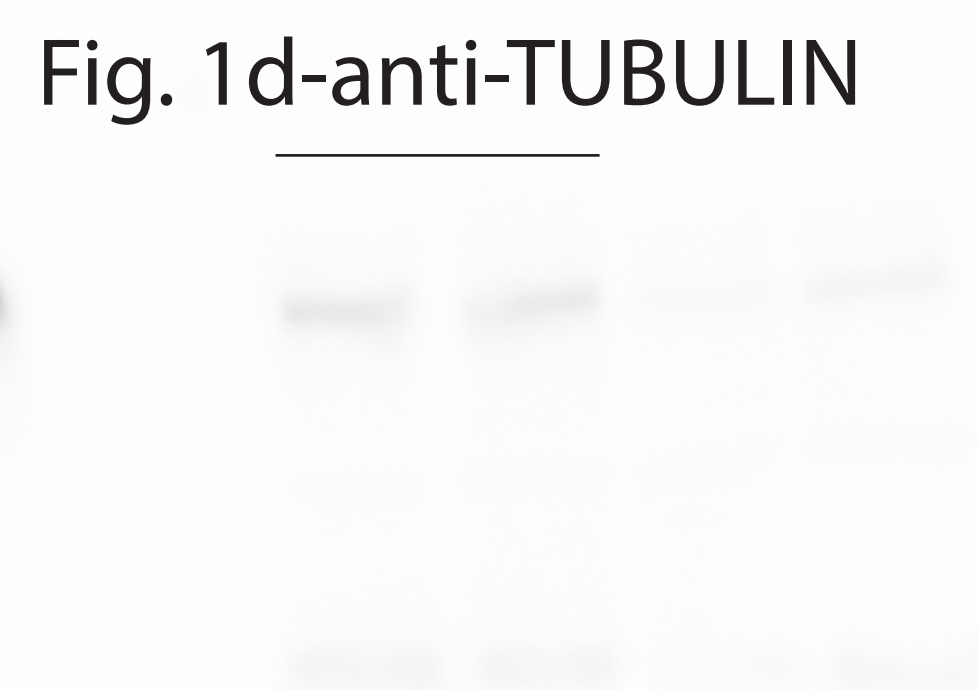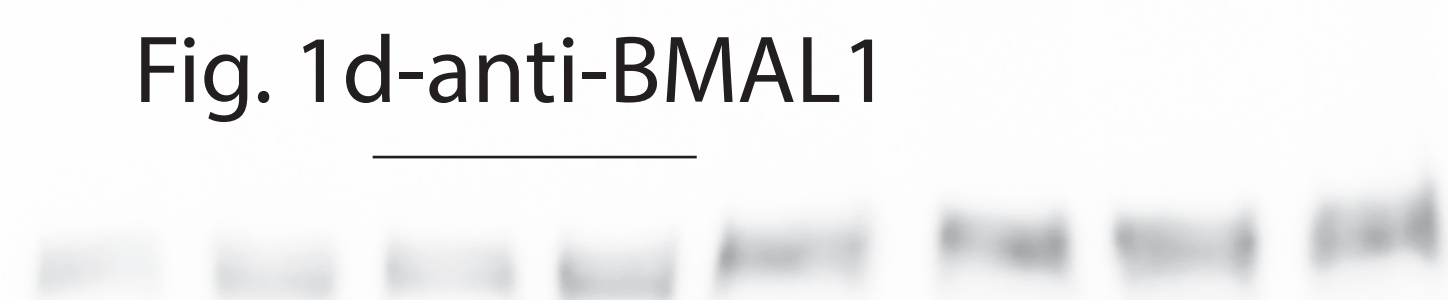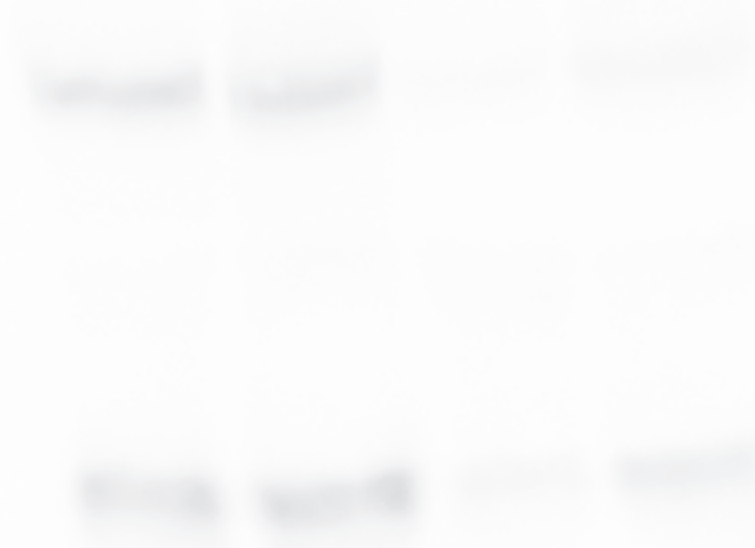

Supplement: Supplementary file 8 — Source Data [file 41467_2021_26567_MOESM8_ESM.zip › Source Data/Fig.1d_western_blot_BMAL1&TUBULIN.pdf]

Fig. 1d-anti-HNF4A

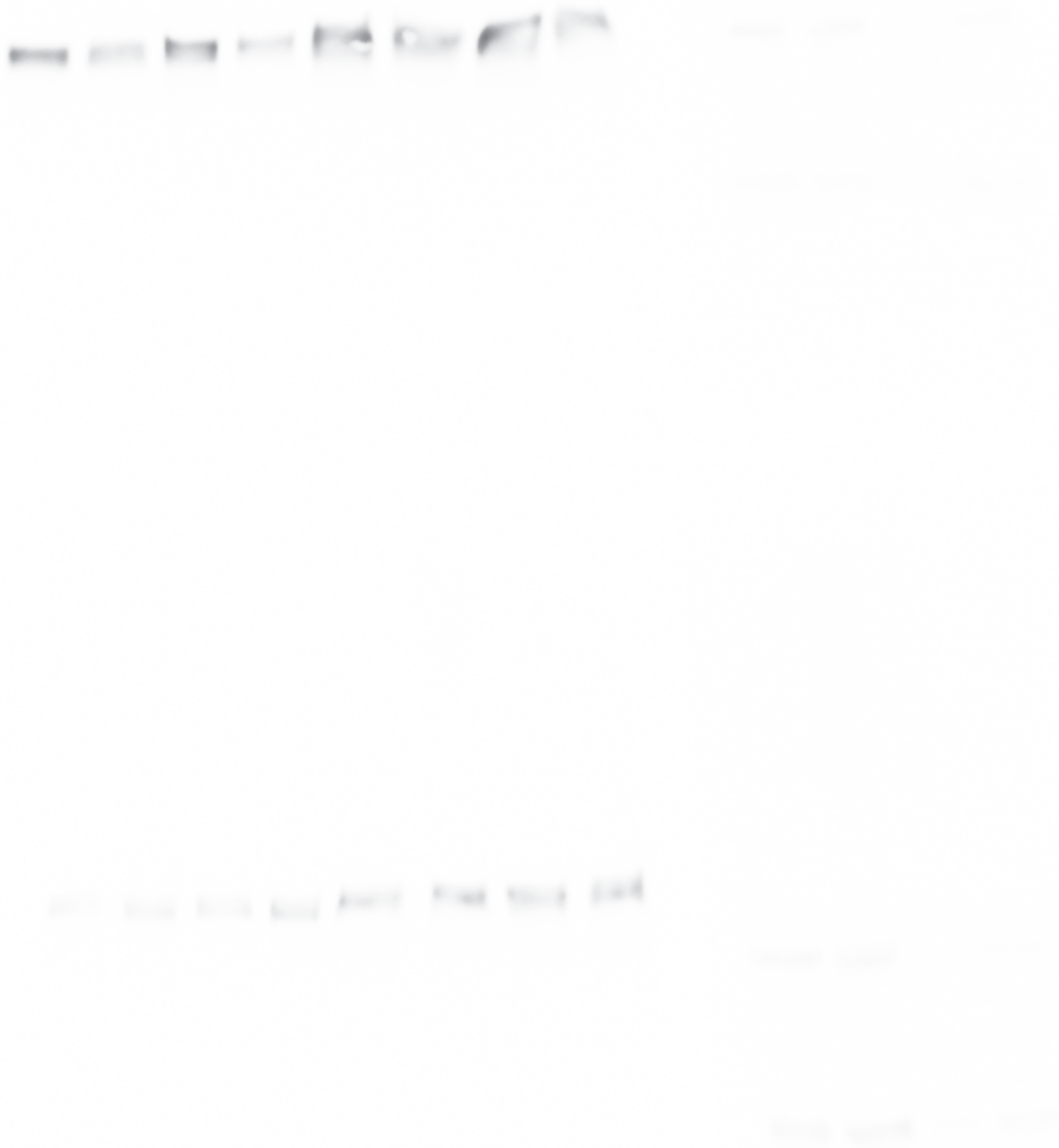

Supplement: Supplementary file 8 — Source Data [file 41467_2021_26567_MOESM8_ESM.zip › Source Data/Fig.1d_western_blot_HNF4A.pdf]
